# Local and global trace plutonium contributions in fast breeder legacy soils

**DOI:** 10.1038/s41467-021-21575-9

**Published:** 2021-03-19

**Authors:** Chris Tighe, Maxi Castrillejo, Marcus Christl, Claude Degueldre, Jeremy Andrew, Kirk T. Semple, Malcolm J. Joyce

**Affiliations:** 1 Department of Engineering, Lancaster University, Lancaster, UK; 2 Laboratory of Ion Beam Physics, ETH - Zürich, Zürich, Switzerland; 3 Dounreay Site Restoration Ltd., Dounreay, Thurso, Scotland; 4 Lancaster Environment Centre, Lancaster University, Lancaster, UK

**Keywords:** Environmental impact, Environmental monitoring, Nuclear waste

## Abstract

Trace-level plutonium in the environment often comprises local and global contributions, and is usually anthropogenic in origin. Here, we report estimates of local and global contributions to trace-level plutonium in soil from a former, fast-breeder reactor site. The measured ^240^Pu/^239^Pu ratio is anomalously low, as per the reduced ^240^Pu yield expected in plutonium bred with fast neutrons. Anomalies in plutonium concentration and isotopic ratio suggest forensic insight into specific activities on site, such as clean-up or structural change. Local and global ^239^Pu contributions on-site are estimated at (34 ± 1)% and (66 ± 3)%, respectively, with mass concentrations of (183 ± 6) fg g^−1^ and (362 ± 13) fg g^−1^. The latter is consistent with levels at undisturbed and distant sites, (384 ± 44) fg g^−1^, where no local contribution is expected. The ^240^Pu/^239^Pu ratio for site-derived material is estimated at 0.05 ± 0.04. Our study demonstrates the multi-faceted potential of trace plutonium assay to inform clean-up strategies of fast breeder legacies.

## Abstract (web summary)

This study proposes a method to differentiate between local plutonium-based contamination in soils versus trace plutonium stemming from global dispersion in the past, such as fallout from detonation and atmospheric testing of nuclear weapons.

## Introduction

Minute quantities of plutonium occur naturally in the terrestrial environment^1^ from neutron capture on ^238^U in uranium-rich deposits. Anthropogenic contributions occur local to their source of production, for example, from nuclear plant effluents, reactor accidents, accidents involving nuclear weapons and plutonium-powered space probes etc., and also globally from fallout. Following the measurements Seaborg et al.^1,2^ of Canadian pitchblende (yielding a ^239^Pu mass concentration of 550 fg g^−1^), Krey et al.^3^ estimated the ^240/239^Pu ratio due to fallout, at 0.176 ± 0.02: this has been used widely for comparison by subsequent studies. Harley^4^ suggested a distribution of the oxide from weapons testing of 2 mCi km^−2^ (~485 fg g^−1^ assuming deposition in the first 5 cm and a bulk soil density of 1.33 g cm^−3^) in the north temperate zone. The depth-resolved assessment of plutonium residues in Lake Ontario sediments followed, yielding a ^240^Pu/^239^Pu ratio^5,6^ consistent with Krey et al. Subsequently, Buesseler et al.^7^ reported measurements of a dated coral record from the North Pacific of ^239^Pu mass (~55 fg g^−1^) and ^240/239^Pu ratio (>0.2), the latter reflecting the predominance of fallout in this environment, in agreement with the prior art^3^. Plutonium-239 abundance of the contrasting residues from the Cigar Lake natural ore body (Canada) were reported^8^, resolved with depth, in the range 17–6200 fg g^−1^. Kelley et al.^9^ reported on the global distribution, including a sample from Wick (Scotland), ~50 km from the site at Dounreay studied in this research, with a ^240^Pu/^239^Pu ratio of 0.182 ± 0.001. Muramatsu et al. published an analysis of samples from the Marshall islands^10^ with a ^240^Pu/^239^Pu range of 0.065–0.306 and ^239^Pu mass 75–500 fg g^−1^. In 2013, Steier et al.^11^ reported accelerator mass spectrometry (AMS) measurements of samples from Sellafield (UK) with a ^240^Pu/^239^Pu range of 0.183–0.228 (±0.001) and, in 2015, Armstrong et al.^12^ reported a 10-year assessment of samples from Savannah River (USA) with ^240^Pu/^239^Pu range of 0.0757–0.3035.

Terrestrial plutonium abundance, albeit deposited over decades, varies slowly with time due to the long half-lives of most plutonium isotopes and relatively slow natural transport mechanisms, in the absence of human intervention. The ability to discern local sources of plutonium from global fallout is important to inform decisions concerning nuclear legacies, particularly the clean-up of contaminated land. As Harley^4^ stated, it remains ‘…desirable…to distinguish…local sources from global fallout’ for exactly this reason. Given this context, the motivation for this study is to determine whether the local contribution to trace plutonium abundance on a fast breeder site might be discerned from the global contribution, to inform the extent to which clean-up of the local component might be necessary.

In addition to the variation in trace elemental plutonium, the isotopic distribution varies too. Plutonium-239 is the more probable product of activation in reactors relative to the higher-mass plutonium isotopes. Industrially, fissile Pu, in particular ^239^Pu, has also been the focus of nuclear reprocessing, mixed-oxide and metal fuel manufacture, and nuclear weapons fabrication operations for ~70 years worldwide, albeit on a localised basis. Of particular relevance to this research is ^239^Pu produced by breeding in the mid-to-late 20th century and the legacy of these activities.

In comparison with ^239^Pu, the higher-mass plutonium isotopes (^240^Pu, ^241^Pu, ^242^Pu and ^244^Pu) have few specific applications and have not been manufactured on a comparable scale. Since their formation is dependent on the neutron fluence and energy spectrum, estimating the extent to which they are produced can require knowledge of the associated neutron environment, in which they are made. Elemental plutonium concentration in soils is dependent on many factors, in addition to the way in which it was formed, such as geographic location, precipitation and soil composition etc. By contrast, the link between a specific isotope, considered via the isotopic ratio, and its origin often yields a more tangible association^13^, for example: a pre-detonation weapon safety test might cause ^239^Pu to dominate; fallout from atmospheric detonations can comprise higher-mass isotopes through to ^244^Pu (refs. ^14,15^), whereas a relatively low ^240^Pu/^239^Pu ratio might reflect the low proportions of ^240^Pu desirable in some applications, such as that obtained by breeding. Material dispersed in fallout will often have a higher proportion of ^244^Pu (ref. ^16^), whereas higher-burnup manufacture in thermal spectrum nuclear reactors, might afford an inventory effectively limited to ^240^Pu and ^241^Pu, generally with a higher proportion of ^240^Pu; the decay of ^241^Pu affords for the activation of americium products by neutrons and the formation of curium isotopes^17^, also less prominent in contributions from weapons fallout. While the isotopic association, particularly to identify plutonium in fallout, is well understood we are not aware of its use to discern the local contribution to breeder-borne material in the environment: hence the focus of this study.

Trace plutonium can be estimated by α-assay of environmental samples, e.g., calcined shellfish or leached terrestrial minerals, etc. and γ-ray spectrometry can be used to infer trace ^241^Pu levels in environmental samples^18^, but the relevant lines for the other isotopes can be too weak. Alternatively, detection limits achieved with sector-field ICP-MS have advanced significantly of late providing, for example, trace isotopic plutonium evidence associated with Fukushima^19,20^. To discern isotopics at trace levels AMS has been used widely^21,22^. Fifield et al.^23–25^ suggest a limit of detection for ^239^Pu two orders of magnitude lower for AMS than for α spectrometry.

Plutonium breeding aims to achieve a positive yield (in particular of ^239^Pu) relative to the amount of fissile material consumed. A fast neutron spectrum is required to achieve the neutron multiplicities necessary to offset neutron losses due to absorption and leakage. Originally, plutonium breeding^26^ constituted a closed fuel cycle unhindered by what was forecast to be the relatively limited abundance of ^235^U. Several major projects followed, for example: the Prototype Fast Reactor at Dounreay (UK)^27^, Phénix (France)^28^, KNK II (Germany)^29^, FBTR (India)^30^, Jōyō (Japan)^31^, BN-600 (Russia)^32^ and the EBR-2 (USA)^33^. Although interest in breeding declined, when global uranium abundance proved higher and consumption proved lower than forecast, a number of the next-generation, Generation IV, reactor designs are breeders^34^.

A principal concern with the clean-up of legacy breeder sites is pollution by plutonium. Past mistakes and the legacy of what was accepted practice has resulted in releases of plutonium to the natural environment. For example, in the context of Dounreay, it is known that contamination from operations will be present in the local environment: there will be contamination from authorised discharges (such as from the gaseous discharge stacks) permitted under the various authorisations and permits that the site has operated under. There are also expected to be some localised spots of contamination arising from historical movement of materials around site. In contrast with dispersed contamination on-site, individual particles arose at Dounreay as a result of an accidental discharge at sea. These have a very different isotopic composition consistent with irradiation in the materials test reactor^35^, and arose on the seabed and occasionally on beaches, in contrast to the dispersion of material from the gaseous discharge stacks on the site itself described earlier, which is studied in this work.

**Fig. 1:**
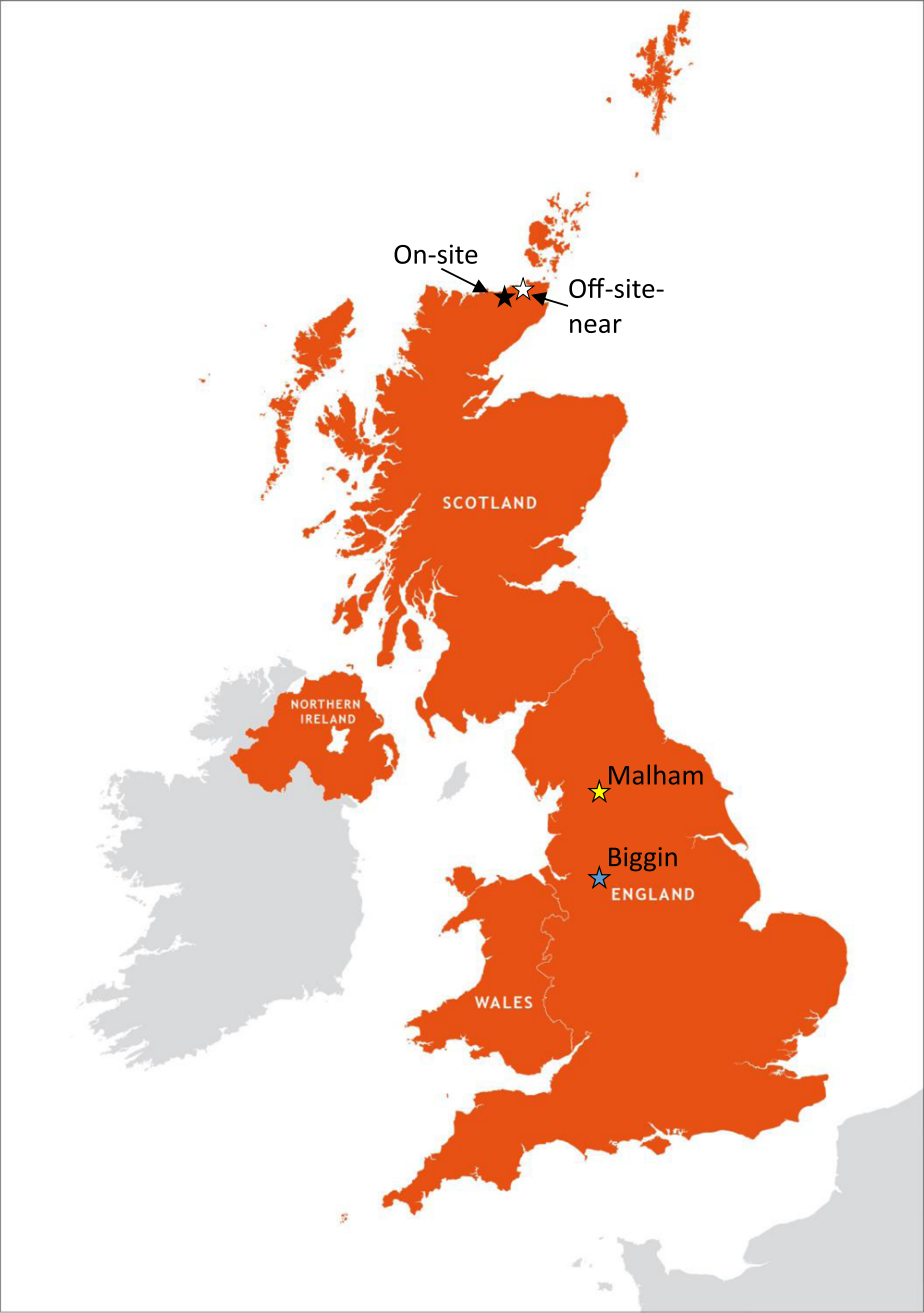
A map of the United Kingdom with sample locations. On-site samples are denoted with a black star; off-site-near: white star; Malham: yellow star; Biggin: blue star. Base map © Maproom.

Twelve soil samples were collected on-site (nine samples), near to (off-site-near, one sample) and far away (off-site-far, 2 samples) from Dounreay, near Thurso, on the north coast of Scotland. The nine samples from Dounreay were collected from a 220-m long invasive trench excavation of 1 m depth (assuming 0.5 m average depth after random sampling). Off-site samples include: an off-site-near sample taken near to the Dounreay site but not on it, to cater for local variations due to dispersion of material from activities on-site beyond site boundaries, fallout in the local area and uranium-rich geological deposits in the area. Two off-site-far sources of samples were taken off-site 700 km to the south: Malham in Yorkshire and Biggin in Derbyshire, both UK. These were taken from locations exhibiting no evidence of having been disturbed and that are isolated from industrial activity. Approximately 5 kg of soil was collected per sample; each was oven dried and ground to ensure a uniform and homogenous grain size distribution prior to preparation for 5 g per sample being used for measurement by AMS. A map indicating the locations from which the samples were taken is given in Fig. 1.

Here, we report estimates of the local and global contributions to trace-level plutonium in soil samples from the former fast-breeder reactor site at Dounreay, UK, made with AMS. The average ^240^Pu/^239^Pu ratio on-site is observed to be anomalously low, relative to the global average, but qualitatively consistent with the reduced yield of ^240^Pu expected in plutonium bred with a fast neutron spectrum. We observe anomalies in plutonium concentration and ^240^Pu/^239^Pu ratio associated with two on-site samples, which suggest the potential to provide forensic insight into actions on-site at some point in the past, such as localised sites of contamination or the removal of buildings. Local and global contributions to ^239^Pu on-site are estimated at (34 ± 1)% and (66 ± 3)%, respectively, with mass concentrations of (183 ± 6) fg g^−1^ and (362 ± 13) fg g^−1^. The latter is consistent with our measurements of the average at undisturbed and distant sites (384 ± 44 fg g^−1^), where no local contribution is expected. We compare our measurement of the site average ^240^Pu/^239^Pu with that of one of the local anomalies to determine the ratio for site-derived material, which we estimate to be 0.05 ± 0.04. The novelty in our study is that we apply AMS to soil samples from a fast breeder site, we separate local and global contributions, where the isotopic inventory of the local component is consistent with that derived from a fast breeder, we identify the potential for forensic analysis of two-specific, inventory-based anomalies on-site and we estimate the ^240^Pu/^239^Pu ratio for local material, self consistently, which is observed to be consistent with material of breeder origin.

## Results

### Plutonium concentrations

**Fig. 2:**
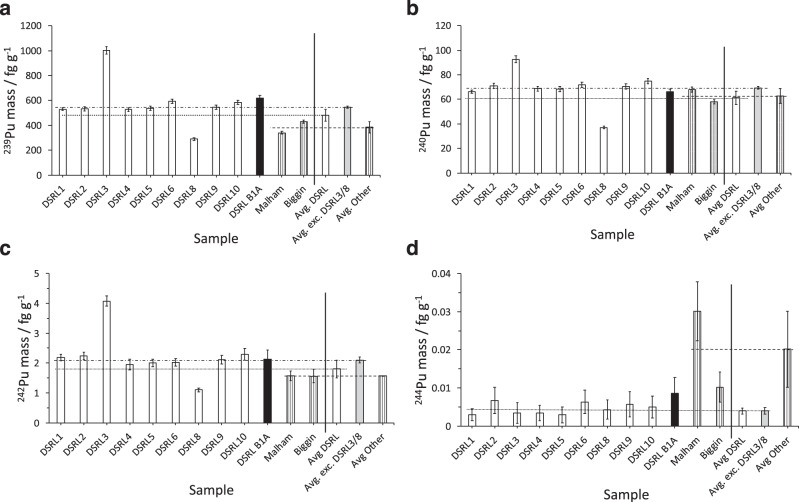
Plutonium mass abundance (fg g^−1^) as a function of sample identifier in soil. **a** ^239^Pu, **b** ^240^Pu, **c** ^242^Pu and **d** ^244^Pu. Uncertainties are depicted by ±1*σ* error bars. Data for samples taken on-site at Dounreay and the corresponding weighted averages (where applicable to the right of the vertical solid line) are depicted as white bars, off-site nearby by black bars and off-site far away by bars with vertical stripes. The horizontal lines represent the weighted average for on-site at Dounreay (dotted), on-site excluding DSRL3 and DSRL8 (dotted-dashed) and off-site-far (dashed). The weighted average for the on-site data excluding samples DSRL3 and DSRL8 is denoted by the shaded grey bar. The corresponding data for weighted averages for on-site, on-site without DSRL3 and DSRL8, off-site nearby, Malham and Biggin are given in Table 1.

AMS results for the concentration of the ^239^Pu, ^240^Pu, ^242^Pu and ^244^Pu isotopes in the samples are given in Fig. 2a–d, respectively. Concentrations are presented with their corresponding weighted averages for samples derived from locations at the Dounreay site, provided by Dounreay Site Restoration Ltd (DSRL), denoted: DSRL1-10, off-site-near, denoted: DSRL B1A and off-site-far, denoted: Malham and Biggin. The corresponding data for the four isotopes are presented in Table 1 (the on-site samples presented in terms of the weighted average of the samples assessed, with the error in the mean and the associated $\chi _\nu ^2$ for the weighted mean calculation for the on-site samples).

**Table 1:** Weighted average mass concentrations of ^239^Pu, ^240^Pu, ^242^Pu and ^244^Pu per mass of dry soil.

Isotope/mass (fg g^−1^)	On-site weighted average				Off-site near	Malham	Biggin
	Inc. DSRL3/DSRL8	$\chi _\nu ^2$	Exc. DSRL3/DSRL8	$\chi _\nu ^2$			
^239^Pu	481 ± 48	101	545 ± 9	2.3	620 ± 19	341 ± 10	428 ± 13
^240^Pu	61 ± 5	80	69 ± 1	2.3	66 ± 2	68 ± 2	58 ± 2
^242^Pu	1.8 ± 0.3	43	2.1 ± 0.1	0.7	2.1 ± 0.3	1.6 ± 0.2	1.6 ± 0.2
^244^Pu × 10^−3^	3.9 ± 0.8	0.3	4.0 ± 0.9	0.4	9 ± 4	30 ± 8	10 ± 4

For samples from on-site at Dounreay, off-site but near to Dounreay (off-site-near) and ~700 km distant (off-site-far: Malham and Biggin). Errors quoted are the error of the mean. Data for on-site samples are presented with and without the DSRL3 and DSRL8 outliers and the corresponding $\chi _\nu ^2$.

### Isotopic ratios

**Fig. 3:**
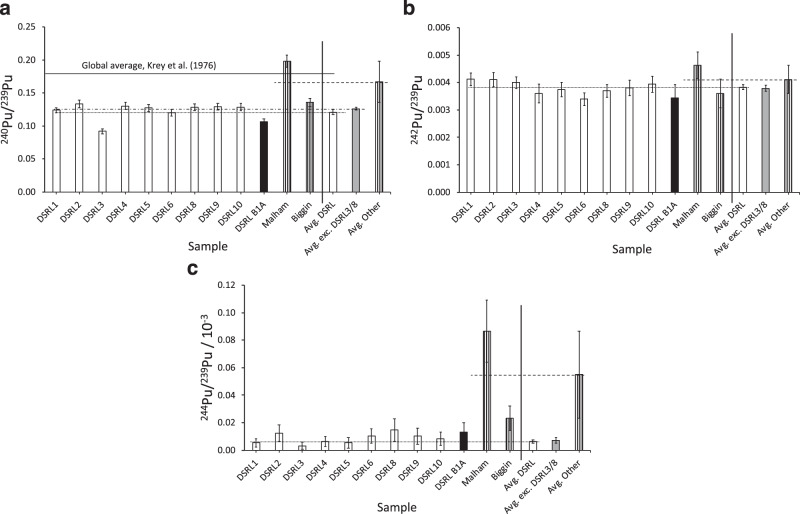
Isotopic ratios for plutonium. **a** ^240^Pu/^239^Pu, **b** ^242^Pu/^239^Pu and **c** ^244^Pu/^239^Pu. Uncertainties are depicted by ±1*σ* error bars. Data for samples taken on-site at Dounreay and the corresponding weighted averages (where applicable to the right of the vertical solid line) are depicted as white bars, the off-site nearby sample by the black bar and off-site far away samples by bars with vertical stripes (the weighted average for the on-site data excluding samples DSRL3 and DSRL8 is denoted by the shaded grey bar). The horizontal lines represent the weighted averages of the ratios on-site at Dounreay (dotted), on-site excluding DSDRL3 and DSRL8 (dotted-dashed, where a difference is apparent) and off-site-far (dashed). The solid horizontal line in **a** corresponds to the global average for the ^240^Pu/^239^Pu ratio reported by Krey et al.^3^, as labelled, of 0.176 ± 0.02. The corresponding data for the weighted averages for on-site, off-site nearby, Malham and Biggin are given in Table 2.

The ^240^Pu/^239^Pu, ^242^Pu/^239^Pu and ^244^Pu/^239^Pu atomic ratios are shown in Fig. 3a–c, respectively. The corresponding data are presented in Table 2: the on-site samples are presented in terms of the weighted average of the samples assessed, with the error in the mean and $\chi _\nu ^2$.

**Table 2:** Weighted average isotopic ratios for (^240^Pu/^239^Pu, ^242^Pu/^239^Pu and ^244^Pu/^239^Pu) for dry soil.

Isotopic ratio	On-site				Off-site near	Malham	Biggin
	Inc. DSRL3/DSRL8	$\chi _\nu ^2$	Exc. DSRL3/DSRL8	$\chi _\nu ^2$			
^240^Pu/^239^Pu	0.121 ± 0.004	7.7	0.126 ± 0.002	0.7	0.107 ± 0.005	0.199 ± 0.008	0.135 ± 0.006
^242^Pu/^239^Pu × 10^−3^	3.83 ± 0.09	1.0	3.8 ± 0.1	1.2	3.4 ± 0.5	4.6 ± 0.5	3.6 ± 0.5
^244^Pu/^239^Pu × 10^−5^	0.6 ± 0.1	0.6	0.7 ± 0.2	0.3	1.3 ± 0.7	9 ± 2	2.3 ± 0.9

For samples from on-site at Dounreay, off-site but near to Dounreay (off-site-near) and ~700 km distant (off-site-far: Malham and Biggin). Errors quoted are the error of the mean. Data for on-site samples are presented with and without the DSRL3 and DSRL8 outliers and the corresponding $\chi _\nu ^2$.

**Fig. 4:**
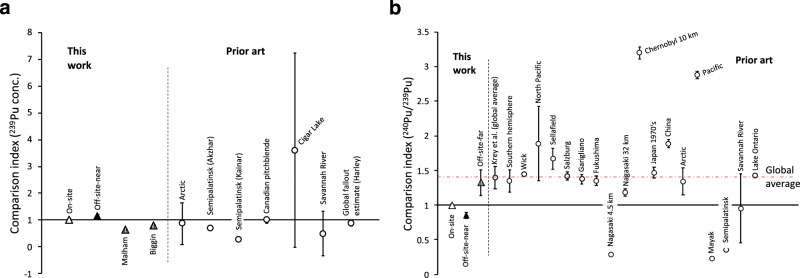
Comparison indices. **a** ^239^Pu concentration and **b** ^240^Pu/^239^Pu ratio, *R*_240/239_. Each comparison is annotated by location, as per, for ^239^Pu concentration: Arctic^13^; Semipalatinsk^37^; Canadian pitchblende^2^; Cigar Lake^8^; Savannah River^12^ and the global fallout estimate^4^. For *R*_240/239_: the global average^3^ (also depicted by the broken red line); the Southern Hemisphere (Mozambique, South Africa, Central Chile and Southern Chile)^48^; Wick^9^; North Pacific^7^; Sellafield, Salzburg and Garigliano^11^; Fukushima and Nagasaki^40^; Chernobyl^49^; 1970’s samples in Japan^50^; China^51^; Pacific^14^ and Mayak^37^. Uncertainties derive from the original works or indicate the standard deviation of concentration indices for the larger sample sets (i.e., Cigar Lake, Nagasaki and Savannah River).

For the different isotopic concentrations presented in Fig. 2a−d, adopting the standard deviation from the mean as a measure of the uncertainty for the DSRL samples (inclusive of DSRL3 and DSRL8 despite these lying outside of the mean of the other on-site samples), assumes no systematic variation between samples. Given the samples originate from different locations on the same relatively small site, one might expect them to be consistent with one another if subject to general sources of plutonium, such as global fallout, material of natural geological origin or a local event, resulting in general contamination of the site. This would also assume that the ground has not been covered or disturbed at some point. By contrast, localised contamination from activities on-site might be expected to vary significantly from one sample location to another. Hence, weighted averages for all samples and for those excluding the outliers (DSRL3 and DSRL8) are provided. Note: only one off-site-near sample was available (DSRL B1A), and hence an average is not possible for this case. Comparisons with the prior art are given in Fig. 4a, b, in terms of a comparison index equal to the ratio of data in previous works to this work for ^239^Pu concentrations and ^240^Pu/^239^Pu ratios, respectively. A complete dataset for concentrations and ratios for each sample is provided in the Supplementary Information.

## Discussion

### Plutonium concentrations

The variance across the ^239^Pu concentration data suggests that the DSRL samples are statistically consistent with one another, except for DSRL3 and DSRL8: DSRL3 has a level greater than neighbouring sample locations from the same site (1001 ± 30 fg g^−1^) and DSRL8 a level that is lower (289 ± 9 fg g^−1^). Including DSRL3 and DSRL8, the weighted average ^239^Pu mass abundance for these samples is (481 ± 48) fg g^−1^. Excluding DSRL3 and DSRL8 from the weighted average yields a $\chi _\nu ^2$ of 2.3, well within the limits (0.17–2.74) defined by a 99% confidence interval for the corresponding number of degrees of freedom, whereas including all samples yields a $\chi _\nu ^2$ of 101—well outside the confidence interval. The weighted average without DSRL3 and DSRL8 is (545 ± 9) fg g^−1^. A similar trend is also exhibited for samples DSRL3 and DSRL8 in the ^240^Pu and ^242^Pu mass concentration data, relative to the concentrations of these isotopes for the other samples, confirming DSRL3 and DSRL8 as outliers.

The off-site-near sample datum for ^239^Pu concentration is consistent with the on-site samples (except DSRL3 and DSRL8), whereas the data for the off-site-far (Malham and Biggin) samples are lower than the on-site average, at (341 ± 10) fg g^−1^ and (428 ± 13) fg g^−1^, respectively. This implies the ambient plutonium level in the Dounreay area may be slightly elevated, relative to Malham and Biggin. Aside from the scenario that this excess derives from legacy activities on-site, it could also be due to (i) increased levels of global fallout in the Dounreay area relative to Malham and Biggin, (ii) additional plutonium consistent with the relatively high indigenous uranium content in the area, 3.00–1470 μg g^−1^ (ref. ^36^), (iii) stronger binding of elemental plutonium to soil media in the Dounreay area due to physicochemical differences between the ground composition of these two, disparate areas, (iv) differences in elevation and rainfall, i.e., given annual precipitation (https://en.climate-data.org/europe/) being higher at Malham (1171 mm, elevation: 213 m) and Biggin (1011 mm, elevation 301 m) relative to that of Caithness (819 mm, elevation: 3 m), of the order ~43% and ~23%, respectively, given some downward migration is expected^4^. The elevation in ^239^Pu is not replicated for ^240^Pu and is at the limit of the uncertainty in the ^242^Pu data.

The ^244^Pu concentrations (Fig. 2d) merit particular mention: unsurprisingly, they are an order of magnitude less than the ^242^Pu data reflecting the greater scarcity of ^244^Pu in terms of yield, notwithstanding its important correlation with global sources of fallout associated with atmospheric weapons tests, for reasons described earlier. All of the data for on-site (DSRL1 through to DSRL10) and off-site-near (DSRL B1A) are consistent with each other and, interestingly, they are consistent with the mass assessment of the sample from Biggin in Derbyshire. The sample from Malham in Yorkshire is high, at (30 ± 8) × 10^−3^ fg g^−1^ relative to the average on-site (3.9 ± 0.8) × 10^−3^ fg g^−1^; given the consistent levels for ^239^Pu, this excess of Malham over Biggin in ^244^Pu can be understood when the isotopic ratios are discussed, which follows below. Malham and Biggin are located at similar elevations, as specified earlier.

Returning to ^239^Pu, given the effect of latitude, rainfall and soil type cannot be accounted for entirely, the comparison index for ^239^Pu concentration (Fig. 4a) has been restricted to prior art for the north temperate zone to focus on areas exposed to fallout and that host industrial facilities with a history of processing actinide materials. Figure 4a illustrates ^239^Pu concentration at Dounreay agrees with prior estimates by Harley^4^ (of the distribution of weapons fallout in the north temperate zone), various sites in the Arctic^13^ (albeit measurements of significant range), of Canadian pitchblende^2^, Cigar Lake^8^ (again recognising the range involved) and Savannah River^12^ (the latter dataset comprises an outlier due to a significant ^240^Pu contribution in 2009, which has been left in this analysis). Comparison with measurements at two villages off-site from Semipalatinsk-21 in Russia^37^ is also included (Kainar and Azkhar), having contributions from the site in that case and global fallout. However, the scatter in this comparison is significant and the range reported in some of the prior art is large. Of particular relevance is that while Malham and Biggin exhibit ^239^Pu levels consistent with global fallout, Fig. 4a demonstrates the slight enrichment in ^239^Pu in trace plutonium in the on- and near-to-site samples.

### Plutonium ratio data

As plutonium concentrations in soils can be influenced by soil properties that can contrast on a very localised basis, the ratios of the plutonium isotopes for each of the samples can yield information complementary to that derived from isotopic mass. Three hypothetical scenarios merit consideration in this regard:

1. All of the plutonium in on-site samples derives from global fallout without any contribution from Dounreay Fast Reactor (DFR) activities. The isotopic composition of this global contribution on-site and near-to-site would therefore be expected to be uniform and consistent with the global average.
2. Dispersed plutonium on-site derives from both global fallout and authorised discharges from stacks on-site (DFR material). The isotopic composition of these two contributions is likely to be different to one another and that of the combination different to the global average (this scenario is perhaps most likely given the trace levels with which we are concerned).
3. The majority of plutonium in the samples derives from local activities with an isotopic composition contrasting to that of scenarios (1) and (2), given an open scenario immune from global deposition is inconceivable.

For scenario (2), where the total plutonium mass is denoted *M* and comprises the global and local contributions *M*_G_ and *M*_L_, respectively, then,

1 $$M = M_{\mathrm{G}} + M_{\mathrm{L}}$$

For scenarios (1) and (3), *M*_L_ = 0 and *M*_G_ = 0, respectively. Where *M*_G_ and *M*_L_ ≠ 0, each component will have a specific isotopic composition,

2a $$M_{\mathrm{G}} = m_{{\mathrm{G}}_{239}} + m_{{\mathrm{G}}_{240}} + m_{{\mathrm{G}}_{241}} + m_{{\mathrm{G}}_{242}} + m_{{\mathrm{G}}_{244}}$$

and,

2b $$M_{\mathrm{L}} = m_{{\mathrm{L}}_{239}} + m_{{\mathrm{L}}_{240}} + m_{{\mathrm{L}}_{241}} + m_{{\mathrm{L}}_{242}} + m_{{\mathrm{L}}_{244}}$$

Since the production of ^244^Pu is more significant in global fallout than in reactors, especially breeders, this component of *M*_L_ (Eq. (2b)) is expected to be smaller than in *M*_G_ (Eq. (2a)). Note: the mass of ^243^Pu is ignored as its half-life is too short to be significant.

With respect to the correlation across samples observed between the mass of ^239^Pu, ^240^Pu and ^242^Pu (Fig. 2a–c), global and local contributions of plutonium cannot be distinguished because the isotopic composition of each of these sources is not known. However, this can be estimated based on the isotopic breeder-blanket compositions available from the literature. Within this convention, the ^240^Pu/^239^Pu ratio (denoted *R*_240/239_) for scenario (2) becomes,

3 $$R_{{\mathrm{240/239}}} = \frac{{m_{{{\mathrm{G}}_{240}}} + m_{{{\mathrm{L}}_{240}}}}}{{m_{{{\mathrm{G}}_{239}}} + m_{{{\mathrm{L}}_{239}}}}}$$

For scenarios (1) and (3), the isotopic ratio is hypothesised to be uniform across samples in each case, and therefore the ratio data (for ^240^Pu, Fig. 3a) for these cases on-site should be flat, given the specific characteristics of ^240^Pu mass (c.f., DSRL3 and DSRL8, Fig. 2b), assuming they are due to disturbance or shelter, would be divided through. While for *R*_242/239_ and *R*_244/239_ this is observed (Fig. 3b, c), in the data for *R*_240/239_ (Fig. 3a), a uniform level is evident for all on-site samples except DSRL3. This sample is low, relative to the other on-site samples (0.092 ± 0.004 apropos the average of the other samples, 0.126 ± 0.002). This is consistent with a local contribution in Eq. (3) present in DSRL3 comprising a high proportion of $m_{{{\mathrm{L}}_{239}}}$ relative to $m_{{{\mathrm{L}}_{240}}}$.

Recapitulating, with reference to DSRL3 and DSRL8, the former is high and the latter is low in terms of ^239^Pu, ^240^Pu and ^242^Pu mass concentration, relative to the averages for the on-site and near-to-site data. However, DSRL3 is low but DSRL8 is consistent with the on-site data for *R*_240/239_. Taken together, these data imply that the proportion of ^239^Pu in DSRL3, relative to ^240^Pu, is higher than for the other samples. A lower ^240^Pu/^239^Pu ratio is expected for material bred specifically to yield ^239^Pu, consistent with DFR activities, and compared with what might be expected from fallout (this is discussed further below with reference to neutron spectra) because ^240^Pu production is lower.

### Overall picture

Concerning DSRL3, the relative ^239^Pu isotopic excess coupled with a relatively low *R*_240/239_ implies a greater association with material from on-site activities rather than fallout, since it is isotopically different to the material adjacent to it. Given the trace levels at which these measurements have been made, it could be associated with, for example, a previously unknown feature or the remnants of a localised case cleared up in the past; suggesting an interesting, localised forensic indication of material likely to be of breeder origin.

DSRL8, by contrast, is low relative to the on-site average for elemental plutonium and, therefore, might be consistent with an area that has been sheltered at some point (i.e., by a structural feature long-since removed) or due to the soil having been removed locally at some point. Since it does not exhibit an isotopic distinction correlating to site activity (in contrast to DSRL3), these hypotheses appear likely to relate to a restriction of the dispersion of elemental plutonium comprising a combination of material of local and global origin.

The data for *R*_244/239_ (Fig. 3c) indicate a consistent picture throughout the on-site, near-to-site and Biggin data. The ratio for the off-site sample from Malham is significantly in excess by an order of magnitude at (9 ± 2) × 10^−5^ compared to the on-site average of (0.6 ± 0.1) × 10^−5^. Since ^244^Pu is the only plutonium isotope not affected by reactor contamination, its lower abundance on-site may indicate the effects of decontamination activities or lower retention in soils on-site etc. A comparison of the ^244^Pu/^239^Pu ratios and the ^244^Pu and ^239^Pu concentrations for on- and near-to-site with far-from-site samples suggests an additional contribution on-site of ^239^Pu and the loss of some ^244^Pu. It is known that the UK falls in a zone where worldwide fallout of radioactivity from nuclear weapons tests exhibits a relatively steep gradient, with increasing northerly latitude^38,39^.

The observation that *R*_240/239_ is consistently low for all of the on-site data, in comparison with both the global average^3^ and the off-site-far data can be appreciated from Fig. 4b in terms of a comparison index (the ratio *R*_240/239_ for the prior art to that for this research). The data from this research fall consistently below the majority of the examples in the prior art associated with fallout, including non-industrial sites nearby (Wick^9^), an industrial site with a relatively long history of discharges from a variety of activities, and thus elevated *R*_240/239_ (Sellafield^11^) and one with a history of weapons-grade plutonium production (Savannah River^12^). In particular, the difference in ratio to Wick (a location near to Dounreay) suggests the difference is a characteristic local to the Dounreay site. Sites synonymous with contamination by material with a very low *R*_240/239_, such as Nagasaki^40^, Semipalatinsk-21 and Mayak^37^ are much lower, as expected.

**Fig. 5:**
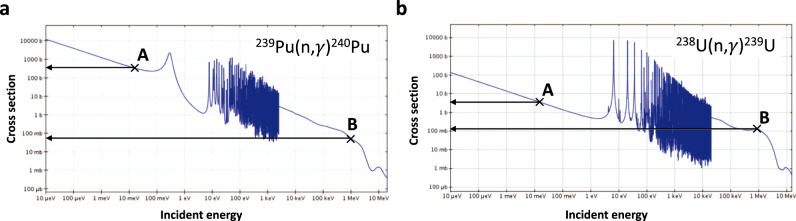
Microscopic neutron cross sections. **a** For the production of ^240^Pu via radiative neutron capture on ^239^Pu, and **b** for the production of ^239^U (and thus ^239^Pu successive β decay) via radiative neutron capture on ^238^U. Both plots have been derived using Janis 4.0 (see: https://www.oecd-nea.org/janis/). The labels ‘A’ denotes the cross section at thermal neutron energies and ‘B’ that at fast neutron energies (~1 MeV) to illustrate the contrast in ^240^Pu yield for fast and thermal neutron energies.

Further corroboration can be drawn with reference to Fig. 5a, b. These depict the microscopic neutron capture cross sections, as a function of neutron energy, for the production of ^240^Pu and ^239^U (and thus ^239^Pu), respectively: in each, ‘A’ denotes the cross section at thermal energies and ‘B’ that at fast (1 MeV) energies. While the need for a fast neutron spectrum in ^239^Pu breeding to yield the neutron economy necessary relative to the thermal case is well known, it is the context with respect to the yield of ^240^Pu that is relevant here: at fast neutron energies (points ‘B’ in Fig. 5a, b), assuming a consistent flux, both ^239^Pu and ^240^Pu have microscopic cross sections of a similar order ~100 mb, whereas at thermal neutron energies (points ‘A’), the microscopic cross sections are significantly different at ~1 b and >100 b, respectively. This illustrates the absence of a thermal neutron contribution in a fast breeder resulting in a reduced yield of ^240^Pu relative to that of a thermal spectrum reactor. It is worthy of note that, while fallout from a detonation would also derive from a neutron spectrum harder than a thermal spectrum fission reactor, it would also have a much higher associated neutron flux, resulting in *R*_240/239_ of a similar order to a thermal spectrum yield^3,41^, and consistent with what is observed in the average of the off-site-far data, i.e., 0.167 ± 0.005. Hence, the lower *R*_240/239_ observed for the on-site samples in this research is consistent with the material comprising a combination of anthropogenic plutonium produced locally by a fast breeder and global fallout, on the basis that less ^240^Pu is produced in a fast breeder relative to that of a thermal spectrum reactor^42^ or fallout, because of the greater thermal neutron component in the former and the higher neutron flux in the latter. It is noted that, while DSRL3 is relatively high in terms of elemental plutonium mass concentration, the *R*_240/239_ for this sample is low, consistent with more of it being derived locally from breeder-borne material, as per this argument. The DSRL3 ratio being low relative to the other on-site samples is consistent with there being a relative excess in breeder material as per the spot contamination scenario suggested above.

### Understanding local and global contributions to ^239^Pu mass

Equation (3) can be rearranged (as shown in the Supplementary Information) to yield an expression for the fraction of the total ^239^Pu mass contribution that is local, $F_{{{\mathrm{L}}_{239}}}$, in terms of the ^240^Pu/^239^Pu isotopic ratios where the total derives from local and global contributions, viz.,

4 $$F_{{{\mathrm{L}}_{239}}} = \frac{{\left( {R_{{\mathrm{240/239}}} - R_{{{{\mathrm{G}}_{240/239}}}}} \right)}}{{\left( {R_{{{{\mathrm{L}}_{240/239}}}} - R_{{{{\mathrm{G}}_{240/239}}}}} \right)}}$$

where *R*_240/239_ is the ^240^Pu/^239^Pu ratio measured in this research on-site at Dounreay, $R_{{{{\mathrm{G}}_{240/239}}}}$ is the global average taken to be that reported by Krey et al.^3^ (0.176 ± 0.02), and the ratio for the local contribution, $R_{{{{\mathrm{L}}_{240/239}}}}$, has been estimated from prior art concerning simulations of the ratio of material bred in an irradiated breeder blanket at (0.03 ± 0.01)^43^. Substitution in Eq. (4) yields an estimate for the local contribution to the trace ^239^Pu abundance on-site of (34 ± 1)% which, using the weighted average for the mass concentration and *R*_240/239_ on-site (comprising both local and global contributions and neglecting the outliers DSRL3 and DSRL8) of (545 ± 9) fg g^−1^ and (0.126 ± 0.002), respectively, suggests local and global contributions of $m_{{{{\mathrm{L}}_{239}}}}$ = (183 ± 6) fg g^−1^ and $m_{{{{\mathrm{G}}_{239}}}}$ = (362 ± 13) fg g^−1^, respectively. The latter estimate is consistent with the average of off-site-far measurements made in this research, (384 ± 44) fg g^−1^, as representing an independent measurement of $m_{{{{\mathrm{G}}_{239}}}}$, suggesting a self-consistent basis on which to discern local and global components in trace plutonium assay.

Alternatively, Eq. (4) can be applied to the specific case of DSRL3, as shown in the Supplementary Material. This assumes it comprises a specific excess of the local ^239^Pu contribution with the same $R_{{{{\mathrm{L}}_{240/239}}}}$ present in lesser quantities for the other on-site samples. Assuming that both local and global contributions, and soil retention and the influence of rainfall are uniform across all on-site samples (save DSRL8), yields a pair of simultaneous equations: one for the site average and one for DSRL3. These yield $m_{{{{\mathrm{L}}_{239}}}}$ = (219 ± 126) fg g^−1^ (40% of the on-site weighted average) and $R_{{{{\mathrm{L}}_{240/239}}}}$ = (0.05 ± 0.04). This approach removes the requirement for an independent estimate of $R_{{{{\mathrm{L}}_{240/239}}}}$ and the potential for uncertainty concerning breeder reactor operating characteristics, while adding credence to the use of the estimate for $R_{{{{\mathrm{L}}_{240/239}}}}$ from Chirayath et al.^43^ above. However, the uncertainties are greater via this route.

AMS has long been known to be capable of class-leading sensitivity with respect to the important requirement of trace actinide assay in the natural environment, and particularly the challenge associated with plutonium contamination. In this research, it has been applied to a comparison of soil samples from a legacy fast breeder site, a sample near to site and those far from site. The research confirms:

- Plutonium-239 concentration at Dounreay agrees with prior estimates of global fallout in the north temperature zone by Harley^4^, at various sites in the Arctic^13^, of Canadian pitchblende^2^, Cigar Lake^8^ (albeit there being a significant range) and Savannah River^12^, notwithstanding the effects of soil type and rainfall unaccounted for. An excess is apparent relative to distant, non-industrial sites in the UK.
- Specific characteristics have been observed that might correlate with structural activities on-site, i.e., the influence of sheltering by structures on-site or localised operations that have removed soil at some point.
- The consistently low ^240^Pu/^239^Pu ratio for on-site and near-to-site samples suggests a forensic correlation consistent with the material expected from breeder activities produced with a fast spectrum to maximise neutron economy, which yields a reduced proportion of ^240^Pu relative to that of ^239^Pu.
- An elemental excess of Pu combined with a low ^240^Pu/^239^Pu ratio relative to neighbouring samples from nearby locations is observed consistent with a spot contamination scenario.
- Estimates of the local ^239^Pu contribution to site activities, and of the associated *R*_240/239_, have been made. The former is consistent with an off-site-far estimate for the global contribution in the UK and the latter is consistent with that expected for breeder material.

### Wider implications

While all fast breeder sites might be subject to local contamination by plutonium at trace levels, the extent to which our findings might be extrapolated will be dependent on the history of activities on a specific site, historical discharge authorisations and local practice. This research is also relevant to contamination scenarios aside from breeding, where the local material might be discerned by an anomalously low *R*_240/239_. Such an incident might involve material dispersed by accident or where trace quantities of material with a ratio distinct from the global average for fallout arises, for example, as part of a forensic investigation.

## Methods

### Sample preparation and Pu radiochemistry

Quantities of 4–5 g of each sample were subject to a simple acid leaching followed by iron co-precipitation and extraction chromatography prior to AMS. Details about the chemical procedures are reported in Chamizo et al.^44^, and Vila-Alfageme et al.^45^. Briefly, the weighed soil sample was placed in a Teflon^TM^ beaker, moistened and spiked with ~30 pg of ^242^Pu. The ^242^Pu spike was kindly provided by the Centro Nacional de Aceleradores, Seville, Spain, and is traceable to the reference material R15-20 supplied by the National Physical Laboratory, United Kingdom. The Pu isotopes were leached from the soil by addition of concentrated Suprapur® nitric acid and hydrogen peroxide in a hotplate following the guidelines in Sakaguchi et al.^46^. The acidic solution was centrifuged and filtered three consecutive times, evaporated to dryness and diluted in 8 M Suprapur® nitric acid. The valences of Pu were adjusted using 3 N sodium nitrite prior to co-precipitation with clean Fe(OH)_3_ to ensure complete extraction of Pu isotopes. The iron precipitates were re-dissolved in 8 M Suprapur® nitric acid and subject to sequential extraction of Pu and other isotopes (e.g., U) using TEVA® and UTEVA® columns (Triskem). The eluate containing the Pu fraction was coprecipitated using 1.25 mg of clean Fe^3+^ solution and evaporated to dryness. All Pu isotopes were converted to the oxide form by heating to 650 °C and mixed with 1.3 mg of niobium prior to pressing in AMS cathodes. Procedural blanks (*n* = 7) prepared with deionized water were processed in the same way as the samples and were included in every analytical batch to assess the background contribution to Pu isotopes. Each of the samples was assessed by AMS at the ETH-TANDY AMS facility, Zürich, Switzerland^47^. For the ^244^Pu analysis, two samples were prepared and assessed for the DSRL1 source of samples, yielding data DSRL1 and DSRL1S2.

## Supplementary information

Supplementary information

## Data Availability

Access to the data presented in this paper can be provided, and for this and any further inquiries about our work please contact the corresponding authors.
