# Supplementary material for: Local and global trace plutonium contributions in fast breeder legacy soils
Source: Nat Commun. 2021 Mar 19;12:1381. doi: 10.1038/s41467-021-21575-9 (PMC7979690; doi:10.1038/s41467-021-21575-9)
Supplement: Supplementary file 1 — Supplementary information [file 41467_2021_21575_MOESM1_ESM.pdf]

Supplementary Table 1

Sample group		On-site									Off-site	Far-from-site	
Sample no.		DSRL1	DSRL2	DSRL3	DSRL4	DSRL5	DSRL6	DSRL8	DSRL9	DSRL10	DSRLB1A	Malham	Biggin
Mass of sample / kg		5.02	5.04	5.03	5.05	5.06	5.01	5.01	5.02	5.01	5.01	5.02	5.02
Conc.	^{239}Pu / fg g^{-1}	530±11	533±16	1001±30	528±16	536±16	595±18	289±9	545±16	583±18	620±19	341±10	428±13
	^{240}Pu / fg g^{-}	66.1±1.4	71.0±2.1	92.7±2.8	68.7±2.1	68.3±2.1	71.9±2.2	37.1±1.1	70.5±2.1	74.8±2.3	66.2±2.0	67.8±2.1	57.9±1.7
	^{242}Pu / fg g^{-}	2.18±0.11	2.23±0.13	4.08±0.18	1.95±0.17	2.01±0.13	2.02±0.12	1.10±0.06	2.11±0.15	2.29±0.19	2.14±0.29	1.58±0.16	1.56±0.22
	^{244}Pu ($\times10^{-3}$) / fg g^{-}	3.0±1.6	6.7±3.4	3.4±2.7	3.4±2.0	2.9±2.1	6.3±3.1	4.3±2.5	5.7±3.3	5.0±2.9	8.5±4.2	30.1±7.8	10.2±3.9
Ratio	^{240}Pu/^{239}Pu	0.124±0.004	0.133±0.006	0.092±0.004	0.130±0.006	0.127±0.005	0.120±0.005	0.128±0.005	0.129±0.005	0.128±0.005	0.106±0.005	0.198±0.009	0.135±0.006
	^{242}Pu/^{239}Pu $\times10^{-3}$	4.1±0.2	4.1±0.3	4.0±0.2	3.6±0.3	3.7±0.3	3.4±0.2	3.7±0.2	3.9±0.3	3.9±0.3	3.4±0.5	4.6±0.5	3.6±0.5
	^{244}Pu/^{239}Pu $\times10^{-3}$	0.005±0.003	0.012±0.006	0.003±0.003	0.006±0.004	0.005±0.004	0.010±0.005	0.015±0.008	0.010±0.006	0.008±0.005	0.013±0.007	0.087±0.023	0.023±0.009

Supplementary note 1

Mass balance derivation to obtain local and global mass concentrations and ^{240}Pu/^{239}Pu ratio

$$R_{240/239} = \frac{m_{G_{240}} + m_{L_{240}}}{m_{G_{239}} + m_{L_{239}}}$$

$$R_{240/239} = \frac{m_{G_{240}}}{m_{G_{239}} + m_{L_{239}}} + \frac{m_{L_{240}}}{m_{G_{239}} + m_{L_{239}}}$$

$$R_{240/239} = \frac{m_{G_{240}}}{m_{G_{239}}}\frac{1}{(1 + m_{L_{239}}/m_{G_{239}})} + \frac{m_{L_{240}}}{m_{L_{239}}}\frac{1}{(m_{G_{239}}/m_{L_{239}} + 1)}$$

$$R_{240/239} = R_{L_{240/239}}\frac{1}{(m_{G_{239}}/m_{L_{239}} + 1)} + R_{G_{240/239}}\frac{1}{(1 + m_{L_{239}}/m_{G_{239}})}$$

$$R_{240/239} = R_{L_{240/239}}\frac{m_{L_{239}}}{(m_{G_{239}} + m_{L_{239}})} + R_{G_{240/239}}\frac{m_{G_{239}}}{(m_{G_{239}} + m_{L_{239}})}$$

Since:

$$\frac{m_{L_{239}}}{(m_{G_{239}} + m_{L_{239}})} + \frac{m_{G_{239}}}{(m_{G_{239}} + m_{L_{239}})} = F_{L_{239}} + F_{G_{239}} = 1$$

$$F_{G_{239}} = 1 - F_{L_{239}}$$

$$R_{240/239} = R_{L_{240/239}}F_{L_{239}} + R_{G_{240/239}}F_{G_{239}}$$

$$R_{240/239} = R_{L_{240/239}}F_{L_{239}} + R_{G_{240/239}}\left(1 - F_{L_{239}}\right)$$

$$R_{240/239} = R_{L_{240/239}}F_{L_{239}} + R_{G_{240/239}} - R_{G_{240/239}}F_{L_{239}}$$

$$R_{240/239} - R_{G_{240/239}} = F_{L_{239}}(R_{L_{240/239}} - R_{G_{240/239}})$$

$$F_{L_{239}} = (R_{240/239} - R_{G_{240/239}})/\,(R_{L_{240/239}} - R_{G_{240/239}})$$

Supplementary note 2

Derivation of simultaneous equations to yield self-consistent estimate of local contributions and associated $^{240}Pu/^{239}Pu$ ratio

From the above:

$$F_{L_{239}} = \frac{(R_{240/239} - R_{G_{240/239}})}{(R_{L_{240/239}} - R_{G_{240/239}})}$$

Re-arranging to isolate the local $^{240}Pu/^{239}Pu$ ratio, $R_{L_{240/239}}$:

$$R_{L_{240/239}} = \frac{R_{240/239} - R_{G_{240/239}}}{F_{L_{239}}} + R_{G_{240/239}}$$

For DSRL3, we define:

$F_{L_{239\_\mathrm{DSRL3}}}$ = the ratio of local ^{239}Pu to the total of the local and global contributions.
$R_{240/239\_\mathrm{DSRL3}}$ = $^{240}Pu/^{239}Pu$ ratio for DSRL3.

For the average of the 8 samples on-site (excluding DSRL3) we define:

$F_{L_{239\_\mathrm{av}}}$ = the ratio of local ^{239}Pu to the total of the local and global contributions.
$R_{240/239\_\mathrm{av}}$ = $^{240}Pu/^{239}Pu$ ratio for the average of the 8 samples.

To give two equations:

$$R_{L_{240/239}} = \frac{R_{240/239\_\mathrm{DSRL3}} - R_{G_{240/239}}}{F_{L_{239\_\mathrm{DSRL3}}}} + R_{G_{240/239}} \quad \text{(S1)}$$

$$R_{L_{240/239}} = \frac{R_{240/239\_\mathrm{av}} - R_{G_{240/239}}}{F_{L_{239\_\mathrm{av}}}} + R_{G_{240/239}} \quad \text{(S2)}$$

Subtracting S2 from S1:

$$\frac{R_{240/239\_\mathrm{DSRL3}} - R_{G_{240/239}}}{F_{L_{239\_\mathrm{DSRL3}}}} = \frac{R_{240/239\_\mathrm{av}} - R_{G_{240/239}}}{F_{L_{239\_\mathrm{av}}}}$$

$F_{L_{239\_\mathrm{DSRL3}}} = m_{L_{239_{\mathrm{DSRL3}}}} / (m_{L_{239_{\mathrm{DSRL3}}}} + m_{G_{239_{\mathrm{DSRL3}}}})$,

where $m_{L_{239_{\mathrm{DSRL3}}}} + m_{G_{239_{\mathrm{DSRL3}}}}$ =(1001±30) fg g^{-1}

$F_{L_{239\_\mathrm{av}}} = m_{L_{239_{\mathrm{av}}}}/(m_{L_{239_{\mathrm{av}}}} + m_{G_{239_{\mathrm{av}}}})$ where $m_{L_{239_{\mathrm{av}}}} + m_{G_{239_{\mathrm{av}}}}$ =(545±9) fg g^{-1}

Postulate that the global contribution is constant across site, thus: $m_{G_{239_{\mathrm{DSRL3}}}} = m_{G_{239_{\mathrm{av}}}}$
Hence: $m_{L_{239_{\mathrm{DSRL3}}}} = m_{L_{239_{\mathrm{av}}}}$ +456, i.e., the difference between ^{239}Pu concentration for DSRL3 and the average on-site exc. DSRL3 and DSRL8, (456±31) fg g^{-1}, gives:

$$\frac{1001(R_{240/239\_\mathrm{DSRL3}} - R_{G_{240/239}})}{m_{L_{239_{\mathrm{av}}}} + 456} = \frac{545(R_{240/239\_\mathrm{av}} - R_{G_{240/239}})}{m_{L_{239_{\mathrm{av}}}}}$$

$$1001 m_{L_{239_{\mathrm{av}}}}(R_{240/239\_\mathrm{DSRL3}} - R_{G_{240/239}}) = 545 m_{L_{239_{\mathrm{av}}}}(R_{240/239\_\mathrm{av}} - R_{G_{240/239}}) + 456 \times 545\,(R_{240/239\_\mathrm{av}} - R_{G_{240/239}})$$

$$m_{L_{239_{\mathrm{av}}}} = \frac{456 \times 545\,(R_{240/239\_\mathrm{av}} - R_{G_{240/239}})}{\left[1001(R_{240/239\_\mathrm{DSRL3}} - R_{G_{240/239}}) - 545(R_{240/239\_\mathrm{av}} - R_{G_{240/239}})\right]}$$

Substituting: $R_{240/239\_\mathrm{DSRL3}}$=0.092±0.004; $R_{240/239\_\mathrm{av}}$=0.126±0.002; $R_{G_{240/239}}$=0.176±0.02 yields:

$m_{L_{239_{\mathrm{av}}}}$ =(219±126) fg g^{-1}, $m_{L_{239_{\mathrm{DSRL3}}}}$=(675±130) fg g^{-1}

Substituting $m_{L_{239_{\mathrm{av}}}}$ back into S2 in terms of $F_{L_{239\_\mathrm{av}}}$:

$R_{L_{240/239}}$=0.05±0.04
